# The subjective well-being effect of public goods provided by village collectives: Evidence from China

**DOI:** 10.1371/journal.pone.0230065

**Published:** 2020-03-11

**Authors:** Lili Li, Zhonggen Zhang, Changluan Fu

**Affiliations:** 1 China Academy of Rural Development, Zhejiang University, Hangzhou, Zhejiang, China; 2 School of Economics and Management, Hangzhou Normal University, Hangzhou, Zhejiang, China; Southeast University, CHINA

## Abstract

Village collectives are important providers of rural public goods in developing countries with dual urban-rural structures. However, few studies have investigated the relationship between the public goods provided by village collectives and the subjective well-being (SWB) of rural residents. This study aims to fill that gap. Based on the 2014 round of China Family Panel Studies (CFPS) survey data, this study estimates an ordered logit model of a SWB function to examine the role of the public goods provided by village collectives. The results indicate that village collectives’ provision of public goods has a significantly positive effect on the SWB of rural residents by promoting the dual growth of household income and consumption. Village collectives’ public expenditures on production, education, and public services also positively affect the SWB of rural residents. The public goods provided by village collectives have a significantly positive effect on the SWB of young and middle-aged rural residents but not on the SWB of elderly rural residents. Finally, rural residents with low levels of education and health obtain more SWB effects than do residents with high levels.

## Introduction

In 2017, in accordance with the requirements for a "prosperous industry, livable ecology, local civilization, effective governance, and rich life", China implemented a rural revitalization strategy, aimed at improving the policy system for the integrated development of urban and rural areas, as well as accelerating the modernization of China’s agriculture and countryside. The fundamental goal of China’s rural revitalization strategy is to enhance rural sustainability, which is important not only in China but also in other developing countries. Many developing countries, including China, are facing important challenges in their economic and social development. The most important issue is resolving the contradiction between people's growing need for a better life and unbalanced and inadequate national development, especially urban-rural development. The ultimate goal of the rural revitalization strategy is to improve rural residents’ lives. As the OECD has pointed out, there is an increasing demand to use well-being as a way to move beyond the classical income-based approach [1]. Economists are exploring the use of SWB as a direct measure of utility [2]. It is necessary to understand the subjective well-being (SWB) effect of rural residents. At the individual level, SWB reflects individuals' satisfaction with their lives, and reflects the quality of the social system in which they are embedded [3]. At the country level, the SWB of rural residents is regarded as an important issue related to the social harmony, stability, and sustainable development of developing countries. The SWB of rural residents has become an international research hotspot in related fields [4]. Scholars have examined the effects of various factors (e.g., government expenditure, work condition, agricultural technology adoption, entrepreneurship, income, expectation) on the SWB of rural residents in developing countries [5–10], in order to find more effective ways to improve it.

The provision of rural public goods is important for realizing rural sustainability, and is also necessary for realizing farmers’ prosperity and improving their SWB. Scholars have been studying the socio-economic effects of rural public goods provision, mainly from the perspectives of income and poverty reduction. For example, [11] found that infrastructure investment in India had a significant effect on agricultural output and led to higher marginal returns on investment in underdeveloped areas, which helped reduce income inequality. [12] found that poor people could obtain better health and education services, cleaner energy, and government-provided protection from infrastructure investment, as well as higher productivity, lower transportation costs, and higher employment. However, [13] found that the poor in rural Vietnam obtained a lower rate of return on education investment and became even poorer. [14] argued that, if not fully integrated with other public investments, rural infrastructure investments were likely to have a negative impact on income distribution and provide more benefits to rich farmers than poor ones. Other scholars have investigated the impact of rural public investment on poverty reduction in Latin America, Africa, and China among other regions and countries (e.g., [15–17]). However, few studies have focused on how rural public goods provision affects the SWB of rural residents, despite the fact that SWB provides a measure of real utility, which can be used to evaluate the actual effect of rural public goods provision with a high degree of accuracy.

It has been found that the provision of public goods differs between urban and rural areas in developing countries with dual urban–rural structures. Governments have been found to provide limited public goods to rural areas, especially in remote mountainous areas, for various reasons [18]. Therefore, villagers’ self-supporting ability is very important. For example, a study of water conservancy construction in Chinese administrative villages in 2008 found that 9.6% of villages received funds from the government, 13.4% of villages received funds from the village collectives, and 6.6% of villages received funds from other sources, whereas 70.4% of villages received no funds, showing a lack of government support for rural infrastructure development in China. These data suggest that funding from village collectives could be seen as an important source for rural infrastructure development. With the strong support of the Chinese government, the burden of public investment at the village level has been decreasing steadily. Of China's rural public goods investment projects, 37.5% were provided by villages themselves between 2000 and 2003; this figure dropped to 33.88% between 2004 and 2007 and then declined to 20.44% between 2008 and 2011 [19]. However, the village collective is still an important provider of rural public goods. More importantly, village collective self-support usually reflects a higher technical efficiency [18–19]. Village collectives are established by rural residents, and village officials are embedded in a social group, which has a positive effect on the provision of rural public goods [20]. Rural residents are embedded in a solid, high-density social network structure wherein interpersonal relationships can significantly promote the provision of rural public goods [21].

This study explores how public goods provided by village collectives affect rural residents in China, with a focus on SWB, in order to fill a gap in the literature and advance our understanding of the consequences of public goods provided by village collectives in developing countries. China, the world's largest developing country with a large rural population, is an important case in this context. Examining China's collective practice for rural public goods can offer both empirical evidence regarding the relationship between public goods provided by village collectives and rural residents' SWB and a China's useful reference for other developing countries. Our results reveal that the public goods provided by village collectives positively affect the SWB of rural residents because they increase household income while also promoting the expansion of household consumption. Further analyses find that three types of village collective public expenditure—production investment, education investment, and public service expenditure—can improve the SWB of rural residents. The public goods provided by village collectives have a significantly positive effect on the SWB of young and middle-aged rural residents. This study highlights the importance and advantages of rural village collectives’ self-support, thus contributing to both the rural public goods field and well-being theories.

The remainder of this paper is organized as follows. Section 2 proposes our conceptual model. The third section explains our data sources and presents the study’s methodological approach. The empirical results are presented and discussed in Section 4. The last section concludes the paper.

## Conceptual model

Well-being has different meanings in different disciplines, leading to heterogeneous definitions. In economics, well-being has been closely associated with utility since the advent of classical economics. [22] argues that the term "utility" encompasses the experiences of pleasure and pain, wherein positive utility means more happiness and negative utility means more pain. Here, there is a relationship between utility and well-being, and well-being is an attribute of utility. Behavioral economists argue that utility can be divided into two aspects: decision utility and experience utility [23]. The former refers to the importance of a choice compared to other choices, which corresponds to the “desire” meaning of utility emphasized by neoclassical scholars, while the latter refers to the happy experience brought by a certain choice, and thus the “happy” meaning of utility emphasized by classical economists. [24] has implicitly suggested that well-being should indicate the satisfaction derived from things. In this definition, well-being and utility are the same concepts, both of which measuring satisfaction. Most modern economic theories analyze "satisfaction" using the concept of utility [25].

The notion of "utility" in modern economics is an abstract concept that describes subjective feelings that vary from person to person, from time to time, and from place to place. Different consumers have different utility assignments or rankings regarding the same item based on their own situation including their own desires and experiences. From the perspective of "satisfaction", “well-being” and “utility” have similar connotations. We thus argue that rural public goods provision contributes to improving the SWB of rural residents and maximizing their utility as reflected in their subjective judgement.

**Fig 1:**
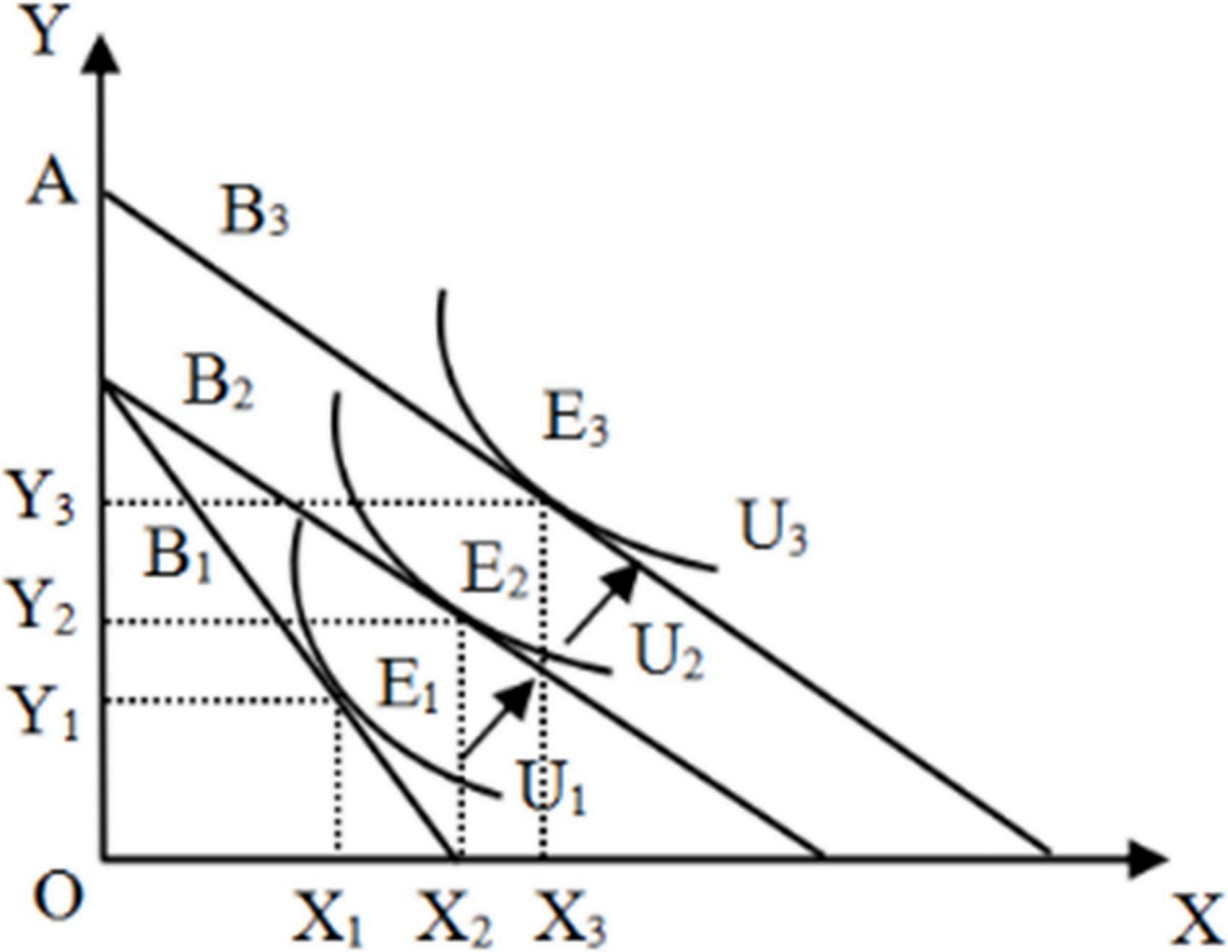
Conceptual model: Village public goods and well-being utility.

Fig 1 depicts the effect of public goods provision on villagers’ well-being utility. It is assumed that the well-being utility *U* of rural residents comes from the consumption set of commodity *X* with public goods attributes and commodity *Y* with private goods attributes. Since commodity *X* has the property of public goods, it is not economic or even expensive for individuals to seek self-satisfaction, and the collective provision of public goods can reduce the cost of obtaining the commodity. Therefore, at a given income level, the indifference curve is tangent to the budget line at E_1_ when the quantities of two goods are *X*_*1*_ and *Y*_*1*_ and the well-being utility is *U* (*X*_*1*_, *Y*_*1*_) = *U*_*1*_. Assuming that villagers display a demand for public goods, as the collective expenditure on public goods increases, the cost for villagers to obtain these goods decreases. In other words, the relative price of goods *X* decreases. The decline in the relative price of commodity *X* will have two effects. On one hand, it will encourage villagers to reduce the consumption of private goods and increase the consumption of public goods (i.e., the "substitution effect"). On the other hand, it will increase villagers’ real income and help them to consume more private goods (i.e., the "income effect"). Since public goods are usually normal goods, the well-being utility level of rural residents will rise from *U*_*1*_ to *U*_*2*_ under the combined effect of the substitution and income effects. In addition, some rural public goods can also help increase villagers’ income. Specifically, productive infrastructure such as roads, electricity, water conservancy, meteorology, and warehousing can directly improve the environment of agricultural production and ensure the stability of agricultural production, thereby reducing agricultural production costs, circulation costs, and transaction costs and thus improving agricultural production efficiency and increasing the income of villagers [26]. Rural infrastructure can also promote the development of non-agricultural industries and increase the non-agricultural income of rural residents. Public education and medical expenditures can enhance the human capital by improving the education level and physical quality of the rural labor force, as well as the incomes of rural residents [27]. As rural residents’ incomes increase, the budget line expands from *B*_*2*_ to *B*_*3*_, and the well-being utility of rural residents increases to the level of *U* (*X*_*3*_, *Y*_*3*_) = *U*_*3*_.

Therefore, the provision of public goods in villages helps to increase the consumption and income of rural residents, thus improving their well-being utility. Theoretically, all types of public goods have positive effects on SWB.

## Data and methods

### Data

The study’s data are derived from national survey data collected in 2014 by China Family Panel Studies (CFPS), a biennial tracking survey conducted by the Institute of Social Science Survey (ISSS) at Peking University. The purpose of CFPS is to investigate China's economic and social development through a nationwide sample survey covering 25 provinces in China. The first-round data collection was carried out in 2010, followed by four additional rounds in 2012, 2014, 2016, and 2018. Community-level data were not included in the surveys carried out in 2012, 2016, and 2018. This study empirically analyzes the effect of the public goods provided by village collectives on the SWB of rural residents using data at the village, household, and individual levels. The measurement method for SWB used in the 2014 questionnaire is different from that used in the 2010 survey. Therefore, we use the 2014 cross-sectional data. Although the names of respondents were included in the CFPS questionnaire, ISSS released the data anonymously.

The study’s dependent variable is the SWB of rural residents. SWB comprises a self-evaluation of both the material quality of life and psychological satisfaction [28]. Thus, it reflects the individual's overall life satisfaction completely and reasonably [4]. Among the several methods of measuring SWB, those that directly collect data on respondent happiness are the most reliable, effective, and comparable [29–30]; thus, many scholars have used this measure. In the CFPS, the question about SWB (which can be found in both the adult and children's questionnaire) is “How happy do you feel?”. The 2014 questionnaire measures SWB on a scale from 0 to 10, from “very unhappy” to “very happy”.

The independent variable is the provision of public goods in the village collectives. The CFPS questionnaire asks three questions on this issue: “How much money was spent on production investment (agricultural and water conservancy, etc.) in your village’s total fiscal expenditure last year?” "How much money was used for public services (roads, water, electricity, gas, sewerage system, etc.) in your village’s total fiscal expenditure last year?” and "How much money was used for investment in education (schools, etc.) in your village’s total fiscal expenditure last year?”; these items deal with the village collectives’ expenditures on production, education, and public services, respectively. This study considers them jointly to establish the total collective expenditure on village public goods, which reflects the level of village public goods provision.

It has been shown that the SWB of rural residents depends on their individual characteristics and family status, and is also influenced by their village environment [31–33]. Therefore, we select variables reflecting resource endowment, economic development, human capital, social capital, and demographic characteristics as control variables, including the per capita agricultural acreage of the village, village non-agricultural output value, village large surname, village resident population, per capita net income of the household, family social capital, whether a family member pays for education, whether a family member works in agriculture, whether a family member works outside the village, gender, age, marriage, education, health, comparison income, and social status.

The 2014 CFPS national sample collected data on 17,883 rural residents in 304 villages across 25 provinces (CFPS does not cover Tibet, Qinghai, Xinjiang, Ningxia, Inner Mongolia, Hainan, Hong Kong, Macau, or Taiwan). After observations with missing values were eliminated, the final sample comprised data on 8,361 rural residents. As shown in Table 1, the average SWB of the villagers in 2014 was 7.36, which reveals that most Chinese rural residents feel that they are happy. The average collective expenditure of village public goods was 1,384,300 yuan in 2014, indicating that, although the government has assumed more responsibility for the provision of rural public goods and the burden on village collectives has been reduced as Chinese government support for the countryside has increased, village collectives still play an important role in rural public goods provision. Expenditures on production, education, and public services in the surveyed villages in 2014 were 590,800 yuan, 76,500 yuan, and 716,900 yuan, respectively. Thus, the expenditures of village collectives are mainly used for public services, followed by production investment. The increased government support for rural education has significantly reduced the education burden on the village collectives. Definitions and descriptive statistics for the control variables are shown in Table 1.

**Table 1:** Variable definition and descriptive statistics.

Variable Name	Definition	Mean	S.D.
*Dependent variable*			
SWB of the villagers	Level of happiness (very unhappy = 0; very happy = 10)	7.36	2.29
*Independent variable*			
Total village expenditure on public goods	Village collective expenditure for public goods provision, unit: 10,000 yuan	138.43	1496.15
Village production investment	Village collective expenditure for production investment, unit: 10,000 yuan	59.08	747.54
Village education investment	Village collective expenditure for investment in education, unit: 10,000 yuan	7.65	77.09
Village public service expenditure	Village collective expenditure for public services, unit: 10,000 yuan	71.69	747.68
*Control variable*			
Per capita agricultural acreage of the village	Unit: mu (1 mu ≈ 0.067 hectares)	2.06	2.23
Village non-agricultural output value	Unit: 10,000 yuan	430.20	1083.30
Village large surname	More than 10% of the households have a large surname = 1, no = 0	0.96	0.18
Village resident population	Unit: person	1955.23	1441.07
Per capita net household income	Unit: yuan	9782.93	10793.23
Family social capital	Family gift expenditure, unit: yuan	3136.29	5093.05
Whether a family member pays for education	Yes = 1, no = 0	0.53	0.50
Whether a family member works in agriculture	Yes = 1, no = 0	0.85	0.35
Whether a family member works outside the village	Yes = 1, no = 0	0.57	0.50
Gender	Male = 1, female = 0	0.51	0.50
Age	Unit: years old	47.36	15.50
Marriage	Yes = 1, no = 0	0.91	0.29
Education	Unit: years	6.08	4.33
Health	Healthy = 3, general = 2, unhealthy = 1	2.48	0.79
Comparison income	Level of comparison income (very low = 1; very high = 5)	2.59	1.03
Social status	Level of social status (very low = 1; very high = 5)	3.06	1.01

S.D. refers to standard deviation.

Yuan is the Chinese currency.

## Methods

As mentioned, the SWB of rural residents is measured from 0 to 10 as an ordinal categorical variable; thus, it is appropriate to use the ordered logit model for empirical analysis. The SWB of rural residents is the dependent variable *Y*, which is an ordered variable with *k* levels (*k* = 11 in this paper). *X^T^* = (*x*_1_,*x*_2_,⋯,*x_n_*) is the independent variable matrix, including core explanatory variables and control variables. *P*(*y = j|x*) is the probability of rank *j* (*j* = 1,2,⋯,*k*). The cumulative probability of rank greater than or equal to *j* (*j* = 1,2,⋯,*k*) is as follows:

P(y≥j|x)=P(y=j|x)+⋯+P(y=k|x) (1)

Further, we can transform this equation into the following logit equation:

$$LogitP_{j}=Logit\left[P(y\geq j|x)\right]=\ln\frac{P(y\geq j|x)}{1-P(y\geq j|x)}\;(j=1,2,\cdots ,k-1)$$ (2)

The ordered logit model is defined as

$$LogitP_{j}=Logit\left[P(y\geq j|x)\right]=‐\alpha_{j}+\sum_{j=1}^{n}\beta_{j}x_{i}\;(j=1,2,\cdots ,k-1;i=1,2,\cdots ,n)$$ (3)

This is equivalent to

$$P(y\geq j|x)=\frac{1}{1+\exp(‐\alpha_{j}+\sum_{j=1}^{n}\beta_{j}x_{i})}\;(j=1,2,\cdots ,k-1;i=1,2,\cdots ,n)$$ (4)

An important application of the ordered logit model is the odds ratio (OR), which is the ratio of the occurrence probability of an event to its non-occurrence probability. It is used to determine how much more likely a particular event is to occur in one group than in another. In addition, we cluster the regression standard error at the household level considering the correlation between the random disturbance items of different members in the same household.

## Results and discussion

### Basic regression results

Table 2 shows the regression results concerning the effect of total village expenditure on the SWB of rural residents. Models 1 and 2 employ the ordered logit model with robust standard errors clustered at the household and village levels, respectively. We find that the correlation within members in the same household is stronger than that within members in the same village. In addition, the premise of using the clustered robust standard error is that the number of individuals in the cluster is small, while the number of clusters is large, and even infinite. In this case, the clustered robust standard error is a consistent estimation of the true standard error. Compared with the clustering on village, the clustering on household reduces the number of individuals and increases the number of clusters. Therefore, we focus on the clustering on household (Tables 3 to 6 report only the estimation results of the clustering on household). As shown in Table 2, Models 1 and 2 both show that the total village collective expenditure on public goods has a significantly positive effect on the SWB of rural residents. Specifically, the OR in Model 1 reveals that, for every 1% increase in total village expenditure on public goods, the probability of improving villagers' SWB increases by 1.3%. These empirical results are in line with the theoretical expectations described above. Thus, expenditures on public goods by village collectives in rural China make an important contribution to improving the SWB of rural residents. This not only reveals the importance of strengthening the provision of rural public goods for SWB, but also reflects the effectiveness of villagers' collective action. In many Chinese villages, village collective expenditure is often undertaken by economic organizations such as cooperatives or is financed by the villagers. Therefore, strengthening rural collective economic organizations and promoting the prosperity of rural industries are important for improving rural sustainability. The collective action of rural households for the provision of public goods represents a kind of endogenous development capability among rural communities [34]. A village without this capability will be unable to promote its residents’ SWB. It is fortunate that most rural communities in China have such developmental capabilities, given their positive effect on the SWB of rural residents.

**Table 2:** The effect of total village expenditure on SWB of villagers.

	Model 1	Model 2
**Total village expenditure on public goods**	1.013*** **(0.004)**	1.013** **(0.006)**
Per capita agricultural acreage of the village	1.051*** (0.011)	1.051*** (0.017)
Village non-agricultural output value	1.000 (0.003)	1.000 (0.005)
Village large surname	0.723** (0.101)	0.723** (0.115)
Village resident population	0.936** (0.030)	0.936 (0.047)
Per capita net household income	1.141*** (0.022)	1.141*** (0.024)
Family social capital	1.005* (0.003)	1.005 (0.004)
Whether a family member pays for education	1.033 (0.048)	1.033 (0.053)
Whether a family member works in agriculture	0.938 (0.061)	0.938 (0.069)
Whether a family member works outside the village	0.872*** (0.043)	0.872*** (0.046)
Gender	0.781*** (0.030)	0.781*** (0.032)
Age	1.023 (0.017)	1.023 (0.024)
Marriage	1.168** (0.084)	1.168** (0.092)
Education	1.024*** (0.006)	1.024*** (0.008)
Health	1.488*** (0.045)	1.488*** (0.046)
Comparison income	1.054** (0.027)	1.054* (0.028)
Social status	1.427*** (0.040)	1.427*** (0.046)
N	8361	8361

Models 1 and 2 employ the ordered logit model with robust standard errors clustered at the household and village levels, respectively, and the OR is reported here.

All continuous variables are regressed in logarithmic form.

Standard errors are reported in parentheses.

*** p < 0.01

** p < 0.05

* p < 0.1

**Table 3:** Regression results of pathway examination.

	Model 3	Model 4
**Total village expenditure on public goods**	0.004** **(0.002)**	0.003** **(0.001)**
Control variables	YES	YES
N	7997	7997

The OLS estimation method is used in Models 3 and 4.

The dependent variables in Models 3 and 4 are household’s per capita net income and per capita consumption, respectively.

All continuous variables are regressed in logarithmic form.

Standard errors are reported in parentheses.

*** p < 0.01

** p < 0.05

* p < 0.1

**Table 4:** Regression results for public goods expenditures.

	Model 5	Model 6	Model 7	Model 8
Village expenditure on production	1.003 (0.003)	1.003 (0.003)		
Village expenditure on production × Whether a family member works in agriculture		1.019** (0.008)		
Village expenditure on education			1.009*** (0.004)	
Village expenditure on public services				1.017*** (0.003)
Control variables	YES	YES	YES	YES
N	8361	8361	8361	8361

Ordered logit models are adopted in Models 5 to 8 and the OR is reported here.

All continuous variables are regressed in logarithmic form.

Standard errors are reported in parentheses.

*** p < 0.01

** p < 0.05

* p < 0.1

**Table 5:** Regression results of heterogeneous effects.

	**Age**		
	**16–39**	**40–59**	**≥60**
Total village expenditure on public goods	1.012* (0.006)	1.017*** (0.005)	1.011 (0.007)
Control variables	YES	YES	YES
N	2566	3740	2055
	**Education**		
	**Literate and semi-literate**	**Primary school**	**Junior high school and above**
Total village expenditure on public goods	1.014** (0.006)	1.017***(0.007)	1.011** (0.005)
Control variables	YES	YES	YES
N	2231	2150	3980
	**Health**		
	**Healthy**	**General**	**Unhealthy**
Total village expenditure on public goods	1.009** (0.004)	1.018** (0.009)	1.024*** (0.008)
Control variables	YES	YES	YES
N	5617	1167	1577

The OR of the ordered logit model is reported here.

All continuous variables are regressed in logarithmic form.

Standard errors are reported in parentheses.

*** p < 0.01

** p < 0.05

* p < 0.1

**Table 6:** Results of instrumental variable regression.

	Model 9	
	First-stage regression	IV regression
Age of the village director	-1.203*** (0.088)	
Whether the village director had run an enterprise	0.716***(0.193)	
Total village expenditure on public goods		0.055* (0.030)
Control variables	YES	YES
F-statistics	38.15	
N	5892	5892

The 2SLS estimation method is used in Model 9.

All continuous variables are regressed in logarithmic form.

Standard errors are reported in parentheses.

*** p < 0.01

** p < 0.05

* p < 0.1

In Model 1, 12 variables—per capita agricultural acreage of the village, village large surname, village resident population, per capita net income of the household, family social capital, whether a family member works outside the village, gender, marriage, education, health, comparison income, and social status—significantly affect the SWB of rural resident. In general, higher villager SWB is associated with larger per capita agricultural acreage in the village, a smaller percentage of villagers with large surnames, a smaller village resident population, higher per capita net household income, higher family social capital, fewer family members working outside of the village, higher education level, higher health status, great comparison income, and higher social status. Female and married villagers tend to be more happy than male and unmarried villagers. This result is consistent with prior research findings. It has been generally confirmed that income, health, education, marriage, and resources are basic guarantees of SWB [35–38]. The empirical results also show that the long-term separation and left-behind problems caused when family members work outside the village are critical obstacles to the SWB of rural residents.

### Pathway examination

As mentioned, the public goods provision of village collectives helps to improve the well-being utility of rural residents via two channels: the income effect and the consumption effect. On one hand, the provision of public goods in villages can increase rural household incomes, thus improving the SWB of rural residents. The provision of public goods can also reduce certain costs and directly increase rural household incomes. For example, improving public facilities such as irrigation and meteorology can reduce the losses caused by natural disasters and ensure the sustainability of agricultural production. Improving agricultural storage, transportation, and other facilities can also reduce the circulation and transaction costs of agricultural products and even increase their added value. The effective provision of rural public goods not only improves farmers' production and living conditions but also promotes the development of non-agricultural industries, which creates more employment opportunities and enhances rural household incomes. For example, public education and medical security indirectly increase the income-generating capacity of rural residents by improving their human capital. On the other hand, the public goods provided by village collectives can promote the consumption level of rural households, thus improving their SWB. Increasing incomes is important for improving farmers' lives, but the effect of rural public goods on rural family life is better reflected by changes in rural household consumption. A substitution effect occurs between the consumption of public goods and the consumption of private goods. The public goods provided by village collectives offer more economies of scale than the individuals' self-provision can offer; this can reduce households’ expenditures on public goods and increase their consumption of private goods. In addition, the provision of productive public goods in rural areas can improve rural production environment and increase farmers’ incomes, thus indirectly affecting their consumption level.

Table 3 shows the OLS regression results regarding the effect of total village expenditures on the incomes and consumption of rural households. The per capita net income and per capita consumption of rural households are the dependent variables in Models 3 and 4, respectively. Table 3 shows that the total expenditure on village public goods has significantly positive effects on both the income and consumption levels of rural households, and their coefficients and significance levels are very close. These empirical results confirm that the improvement of rural residents’ SWB through the provision of public goods stems from the ability of village’s public goods provision to promote the growth of household incomes and consumption capabilities. The increased incomes provide material basis and economic guarantee of SWB for rural residents, and the increased consumption is the ultimate form of improvement in rural family life as it reflects a concrete quality-of-life improvement [39]. In developing countries, increased consumption of rural private goods is accompanied by increased consumption of rural public goods. An insufficient provision of rural public goods will inhibit villagers’ consumption of private goods. Therefore, as the rural economy continues to develop and as farmers' incomes continue to improve, it will be necessary to provide public goods that meet rural demand and thereby enhance consumption in rural areas, transform potential consumption into real consumption, expand the rural consumption market, meet the needs of rural residents, improve their quality of life, and strengthen their SWB.

### Different public goods expenditure

In the CFPS questionnaire, the total expenditure on village public goods is divided into three types: village production investment, village education investment, and village public service expenditure. As mentioned, all types of public goods theoretically have a positive SWB effect. Table 4 shows the regression results regarding how expenditures on different types of public goods affect rural residents’ SWB. Model 5 shows that, as expected, village collective expenditure on production investment has a positive but insignificant impact on villagers’ SWB. When the interaction item reflecting whether a family member works in agriculture was included in Model 6, it was positively significant. This means that whether public expenditure on production can significantly improve villagers’ SWB depends on whether a family member works in agriculture, which is logical and reflects reality. Models 7 and 8 show that village collective expenditures on education investment and public services have a significantly positive impact on villagers' SWB, which is in line with the theoretical expectations. These empirical results show that certain types of public goods are indispensable components of rural public goods and of improving rural residents’ SWB. Fostering SWB among rural residents requires the provision of these types of public goods, so that they can form a strong joint force.

### Heterogeneous effects

The regression results above reflect the average effect of public goods provided by village collectives on the SWB of all rural residents. In reality, however, rural residents consist of various subgroups based on individual characteristics. Few scholars have explored the heterogeneity in the SWB dividends that rural residents receive via the provision of public goods. We checked for intra-group differences in the SWB effect of public goods by conducting a sub-sample regression on the SWB effect of public goods provided by village collectives from the common perspectives of age, education, and health.

As shown in Table 5, the public goods provided by village collectives have significantly positive effects on the SWB of rural residents aged 16 to 39 and 40 to 59 but not on the SWB of rural residents aged 60 and above. This seems to suggest that the public goods provided by village collectives are designed to meet the needs only of those aged 16 to 39 and 40 to 59, while being insufficient for the elderly. On the other hand, it may be that the demand intensity of the young and middle-aged group is lower than that of the elderly; young and middle-aged rural residents may be easily satisfied by the public goods provided by village collectives, while the elderly may be harder to satisfy. China has the largest aging population in the world, especially in rural areas. An aging population brings a host of problems to economic development, such as insufficient pension funds, increased demand for medical benefits, and increased expenditures on health care. Elderly residents have few income sources and their incomes are generally low. They tend to rely on pensions, the levels of which are relatively low. Amid China’s large labor transfer levels, the living conditions of the left-behind elderly have long been a concern. Caring for the elderly group requires not only the efforts of families and communities, but also the support of the government, as well as the participation of non-governmental organizations [40]. The government should bear more responsibility for the provision of public facilities and services to make up for the lack of provision by village collectives [41]. Considering the elderly when investing in rural public goods will increase satisfaction and happiness levels among older people.

Table 5 also shows that the public goods provided by village collectives can significantly improve the SWB of rural residents with different education levels. Comparing the ORs shows that the public goods provided by village collective do better at improving the SWB of villagers who are illiterate or semi-illiterate and those with primary school education than they do at improving the SWB of villagers with junior high school education or above. Rural residents with low education levels are limited by their low skill and income levels, and they rely more on the help of village collectives. The public expenditures of village collectives have improved their chances of increasing their incomes. They have also reduced families’ anxieties about the difficulty in “going to school, seeing a doctor and caring for the elderly”, so that residents dare to spend money and reduce their precautionary savings [42]. Table 5 also shows that the public goods provided by village collectives can significantly improve the SWB of rural residents with different health levels. Comparing the ORs shows that the public goods provided by village collectives do better at improving the SWB of villagers with general and poor health levels than at improving the SWB of healthy villagers. Rural residents in unhealthy and general health conditions value their resources more highly than healthy villagers do. The public goods provided by village collectives can improve their living conditions and bring real benefits to them, which can significantly improve their happiness. When the health level of rural residents increases, the SWB effect of the public goods provided by village collectives weakens. Thus, the SWB of rural residents with low education and health levels is not good at benefiting from private goods but may benefit from public goods well.

### Discussion of endogeneity

A key problem in regression analysis is that the baseline regression results may be endogenous. Theoretically, unobservable factors may affect the SWB of rural residents, resulting in estimation errors caused by missing variables. In addition, a reverse causal relationship may exist between residents’ SWB and village public expenditures: Villagers’ SWB may impact the collective public expenditure of their village, resulting in simultaneous bias. Instrumental variables are the main method of correcting endogenous bias in a model. Several scholars have pointed out that the village director's age and previous entrepreneurial experience can determine the provision of public goods in the village [43–45]. However, the age of the village director and previous entrepreneurial experience do not directly affect villagers’ SWB, which meets the exogenous requirements of instrumental variables. Therefore, we carried out a 2SLS estimation using the age of the village director and whether the village director had run an enterprise before as the instrumental variables for public goods provided by village collectives. Table 6 shows that, in the first-stage regression, both instrumental variables have a significant impact on the total village expenditure on public goods; the F-statistic is 38.15, which is larger than 10 and indicates that the instrumental variables have a strong explanatory power for the endogenous variables. Specifically, the younger the village directors are and the more business experience they have, the more passion and energy they have for managing the village and serving the villagers, and the greater their willingness to invest in public goods through the village collective. Young village directors and those with a business background are generally more extroverted and open, are less conservative, and find it easier to obtain village support and raise funds from villagers [44]. They are also more inclined to use village collective funds as venture capital to invest in village-level public goods. Private business owners and other economic elites have emerged rapidly in the countryside amid the rapid development of China's rural economy. They often run for village cadres to participate in village politics and dominate public power and public decision-making. Amid China’s labor outflow and rural governance decay, the commercial cadres are gradually surpassing the traditional clan network and become the dominant impetus for public goods provision [45]. To examine whether all the instrumental variables are exogenous, we carried out an over-identification test, which accepted the null hypothesis (p = 0.6142). Thus, the two instrumental variables were found to be exogenous and unrelated to the disturbance term. In the second-stage regression, the endogenous explanatory variables containing fit information from the instrumental variables were found to have significantly positive effects on villagers’ SWB. Furthermore, a Durbin-Wu-Hausman test with heteroscedasticity robustness was used to check for endogenous explanatory variables. The results accepted the null hypothesis that all explanatory variables were exogenous (p = 0.1625). Therefore, potential endogeneity problems caused no significant estimation bias in our analysis, and the regression results shown in Tables 2 to 4 are reliable.

## Conclusion and implications

Helping rural residents improve their lives is a fundamental requirement of attaining sustainability in rural areas. China’s rural revitalization strategy aims to remove the obstacles to rural sustainable development and solve the contradiction between the increasing needs of rural residents and the insufficient development of rural areas. An adequate provision of rural public goods is an important component of rural sustainability, and is a necessary condition for rural prosperity and enhance rural SWB. Village collectives are important providers of rural public goods in developing countries with dual urban–rural structures. However, few studies have examined how the public goods provided by village collectives affect rural residents' SWB, even though SWB provides a measure of true utility and reflects the actual effects of rural public goods provision. China’s increased government support for rural development has reduced the burden of public investment at the village level, but the village collective remains an important provider of rural public goods. Using CFPS data, this study empirically analyzes how public goods provision affect the SWB of rural residents using an ordered logit model. The results show that the village collective provision of public goods can improve the SWB of rural residents by increasing rural households’ income levels and consumption capacities. Further analyses find that three types of village collective public expenditure—production investment, education investment, and public service—can have significantly positive effects on the SWB of rural residents. We also find that the public goods provided by village collectives have significantly positive effects on the SWB of young and middle-aged rural residents, across education and health levels, but not on the SWB of elderly rural residents. Moreover, rural residents with low education and health levels obtain more SWB effects from public goods provided by village collectives than do those with high education and health levels.

These findings have important implications for other developing countries that are attempting to promote the collective provision of public goods in villages. Narrowing the gap between urban and rural areas in terms of public goods provision and achieving equal access to urban and rural public services have long been important goals for developing countries, but this is a long-term struggle. We cannot expect public resources to cover all rural areas in the short term. Village collectives must make a difference, and villagers can become self-supporting in some public goods through effective collective actions. China's example shows that being self-supporting in public goods can improve the SWB of rural residents and that we should foster the development of rural collective economic organizations such as cooperatives, expand rural industries, and enhance the economic capacity of village collectives to provide public goods. The provision of village public goods will improve the income and consumption of rural households, thus promoting the further development of the rural economy, forming a virtuous circle. We must also ensure a balanced provision of public goods types, and avoid the short-board phenomenon wherein only certain types of public goods are provided, so that they can form a combined force and significantly improve the SWB of rural residents. Realizing a general and balanced acquisition of SWB dividends from village public goods also requires paying more attention to the elderly and providing them with the public goods and services that meet their needs. Village collective public expenditures have the greatest impact on the SWB of villagers with low education and health levels, showing that village public goods provision is particularly important for such villagers. Village collectives should continue to play a positive role in helping villagers with low education and health levels so as to maximize rural social welfare.
